# Translating Sepsis-3 Criteria in Children: Prognostic Accuracy of Age-Adjusted Quick SOFA Score in Children Visiting the Emergency Department With Suspected Bacterial Infection

**DOI:** 10.3389/fped.2018.00266

**Published:** 2018-10-01

**Authors:** Sietske C. van Nassau, Ron H. van Beek, Gertjan J. Driessen, Jan A. Hazelzet, Herbert M. van Wering, Navin P. Boeddha

**Affiliations:** ^1^Department of Pediatrics, Amphia Hospital, Breda, Netherlands; ^2^Department of Pediatrics, Juliana Children's Hospital, Haga Teaching Hospital, The Hague, Netherlands; ^3^Department of Public Health, Erasmus MC, University Medical Center Rotterdam, Rotterdam, Netherlands; ^4^Intensive Care and Department of Pediatric Surgery, Erasmus MC-Sophia Children's Hospital, University Medical Center Rotterdam, Rotterdam, Netherlands; ^5^Division of Pediatric Infectious Diseases and Immunology, Department of Pediatrics, Erasmus MC-Sophia Children's Hospital, University Medical Center Rotterdam, Rotterdam, Netherlands

**Keywords:** Sepsis-3, (q)SOFA, SIRS, (q)PELOD-2, risk-stratification, prognosis, outcome, pediatrics

## Abstract

**Background:** Recent attempts to translate Sepsis-3 criteria to children have been restricted to PICU patients and did not target children in emergency departments (ED). We assessed the prognostic accuracy of the age-adjusted quick Sequential Organ Failure Assessment score (qSOFA) and compared the performance to SIRS and the quick Pediatric Logistic Organ Dysfunction-2 score (qPELOD-2). We studied whether the addition of lactate (qSOFA-L) would increase prognostic accuracy.

**Methods:** Non-academic, single-center, retrospective study in children visiting the ED and admitted with suspected bacterial infection between March 2013 and January 2018. We defined suspected bacterial infection as initiation of antibiotic therapy within 24 h after ED entry. Age-adjusted qSOFA, SIRS, qPELOD-2, and qSOFA-L scores were compared by area under the receiver operating characteristics curve (AUROC) analysis. Primary outcome measure was PICU transfer and/or mortality and secondary outcome was prolonged hospital length of stay.

**Results:** We included 864 ED visits [474 (55%) male; median age 2.5 years; IQR 9 months-6 years], of which 18 were transferred to a PICU and 6 ended in death [composite outcome PICU transfer and/or mortality; 23 admissions (2.7%)]. 179 (22.2%) admissions resulted in prolonged hospital length of stay. PICU transfer and/or death was present in 22.5% of visits with qSOFA≥2 (*n* = 40) compared to 2.0% of visits with qSOFA<2 (*n* = 444) (*p* < 0.01). qSOFA tends to be the best predictor of PICU transfer and/or mortality (AUROC 0.72 (95% CI, 0.57–0.86) compared to SIRS [0.64 (95% CI, 0.53–0.74), *p* = 0.23] and qPELOD-2 [0.60 (95% CI, 0.45–0.76), *p* = 0.03)]. Prolonged hospital length of stay was poorly predicted by qSOFA (AUROC 0.53, 95% CI 0.46–0.59), SIRS (0.49, 95% CI 0.44–0.54), and qPELOD-2 (0.51, 95%CI 0.45–0.57). qSOFA-L resulted in an AUROC of 0.67 (95% CI, 0.50–0.84) for PICU transfer and/or mortality and an AUROC of 0.56 (95% CI, 0.46–0.67) for prolonged hospital length of stay.

**Conclusion:** The currently proposed bedside risk-stratification tool of Sepsis-3 criteria, qSOFA, shows moderate prognostic accuracy for PICU transfer and/or mortality in children visiting the ED with suspected bacterial infection. The addition of lactate did not improve prognostic accuracy. Future prospective studies in larger ED populations are needed to further determine the utility of the qSOFA score.

## Introduction

As SIRS criteria lack specificity when identifying patients with infection who are at higher risk of mortality, the Adult Sepsis Definition Taskforce published the Third International Consensus Definitions for Sepsis and Septic Shock in 2016 (1). This new Sepsis-3 consensus emphasizes that sepsis can be differentiated from uncomplicated infection by the existence of a dysregulated host response, manifested as hazardous organ dysfunction. The Sequential Organ Failure Assessment (SOFA) score was suggested to be used as a discriminator of in-hospital mortality and has been validated in adult patients with suspected or confirmed infection (1, 2). The quick SOFA (qSOFA) score, incorporating only altered mentation, systolic blood pressure and respiratory rate, has been suggested as manageable bed-side tool to promptly identify infectious patients prone to poor outcomes, and could therefore be especially useful in the Emergency Department (ED) (1, 2). Since publication of the Sepsis-3 consensus, several adult studies in ICU and ED populations have reported that both SOFA- and qSOFA score have better prognostic accuracy compared to formerly used sepsis criteria (2–5).

Regrettably, the Sepsis-3 taskforce excluded pediatric populations from development and validation. Hence, there is a remaining demand for data-driven pediatric sepsis criteria, especially because of pediatric specific challenges in sepsis recognition. Firstly, febrile children present to the ED with milder infections of lower acuity compared to adults. Secondly, pediatric sepsis could have a more fulminant course compared to adults and death could occur very early (6–8), making early recognition even more crucial. Several recent attempts have been made to translate Sepsis-3 criteria to children (9) and although the SOFA score has originally only been validated in patients above 12 years of age (10, 11), age-adapted SOFA and qSOFA show promising results in children admitted to a PICU. Matics and Sanchez-Pinto published a SOFA AUROC of 0.94 for in-hospital mortality (12) and Schlapbach and colleagues reported superior discrimination of age-adjusted SOFA for mortality compared to PELOD-2 and SIRS (13). In the latter, qSOFA performance was slightly better than SIRS, though inferior to SOFA and PELOD-2 scores. A large limitation of these studies is however that they were limited to the PICU, whereas earliest possible recognition of sepsis should occur in the ED.

The aim of this study is to assess the accuracy of the qSOFA score in predicting outcome among children presenting at the ED with suspected bacterial infection. Additionally, we compare our findings with SIRS criteria and the qPELOD-2 score. Lastly, since lactate could be measured in a timely, practically bedside, manner, we hypothesized that the qSOFA score would perform better with lactate included (qSOFA-L) in the risk stratification tool.

## Materials and methods

### Study population

We performed a non-academic single-center retrospective study in patients <18 years who visited the ED and were subsequently admitted to the pediatric ward with suspected bacterial infection between March 2013 and January 2018. We defined suspected bacterial infection as initiation of therapeutic antibiotic therapy within 24 h after ED entry. We considered 11 antibiotics as therapeutic; amoxicillin, amoxicillin clavulanic acid, benzylpenicillin, cefotaxime, ceftazidime, ceftriaxone, cefuroxime, clarithromycin, clindamycin, flucloxacillin, and vancomycin. Patients admitted with a surgical diagnosis were excluded.

### Clinical data collection

Data was retrieved electronically via the hospital patient information system. Data on demographics, antibiotic treatment, vital signs (temperature, heart rate, diastolic, and systolic blood pressure, respiratory rate), laboratory values (lactate, white blood cell count), level of consciousness (AVPU scale, Glasgow Coma Scale) (14), hospital length of stay, PICU transfer and mortality were collected. We calculated four sepsis scores; qSOFA (13), SIRS (15, 16), qPELOD-2 (17, 18), and qSOFA-L (Supplementary Table 1 presents age-adapted scores). These scores were based on the first measured values within 24 h after ED entry. A threshold of two or more points was used to indicate a positive test result for every sepsis score. If only 1 variable was not obtained (i.e., not measured in the first 24 h of admission), we considered this variable to be normal (i.e., no contribution was made to the total score). If two or more variables were not obtained, the total score was considered missing in order to prevent false-negative scores. Cut-off value for lactate was 2mmol/L (19).

### Outcomes measures

The primary outcome measure was a composite of PICU transfer (to an academic, tertiary care center) and/or mortality. Criteria for PICU transfer were cardio-respiratory or neurological failure. The secondary outcome measure was prolonged hospital length of stay, defined as a hospital length of stay of 7 days or longer.

### Statistical analysis

Statistical analyses were performed using SPSS version 24.0 (Armonk, USA). Normality of distribution was assessed through Shapiro-Wilk analysis. Data is presented as percentages, means with standard deviation or medians with ranges, as appropriate. χ^2^-tests were used to compare categorical data by subgroups. We measured the prognostic accuracy of each sepsis score using the area under the receiver operating characteristics curve (AUROC). AUROC comparison was performed using the DeLong method (20) with MedCalc version 18.2.1. Sensitivity, specificity, negative predictive value (NPV) and positive predictive value (PPV) were calculated for each score. *P* < 0.05 were considered statistically significant.

### Ethical aspects

This study was conducted in accordance with the Declaration of Helsinki and Good Clinical Practice guidelines. The study protocol was approved by the local ethical review board (MEC-2018-1063). Necessity for written informed consent was waived.

## Results

### Study population

**Figure 1:**
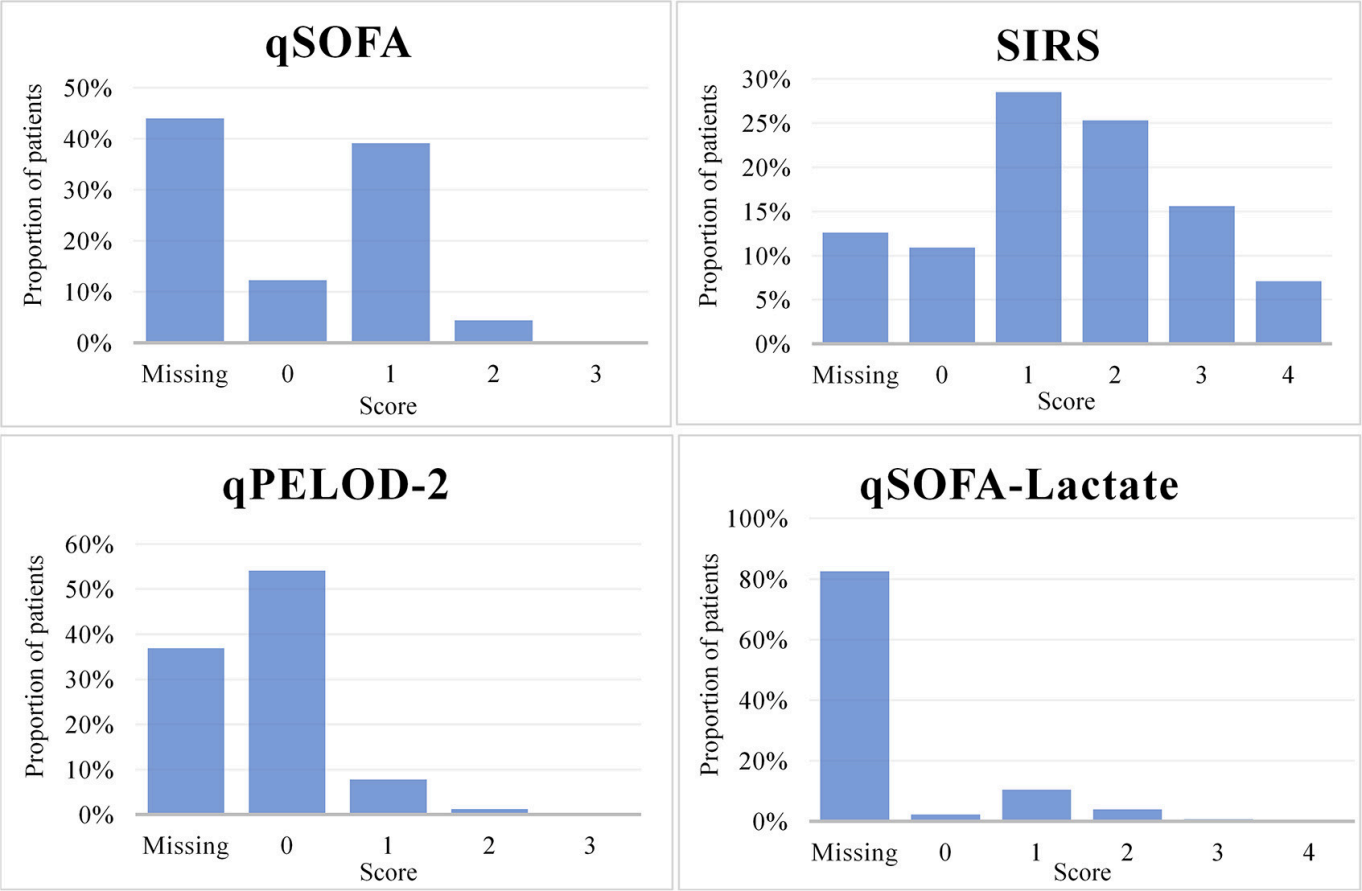
Distribution of qSOFA, SIRS, qPELOD-2, and qSOFA-L scores in pediatric ED encounters with suspected bacterial infection.

We identified 864 ED visits (55% males) with suspected bacterial infection that resulted in admission. Median age was 2.5 years (IQR 9 months-6 years). Six admissions (0.7%) ended in death within 30 days and 18 children (2.1%) were transferred to a PICU. Causes of death were; neurological failure due to cerebral hemophagocytic lymphohistiocytosis (*n* = 1), respiratory failure due to viral infection (*n* = 2) and aspiration pneumonia (*n* = 1), pulmonary artery embolus as a result of ethmoiditis (*n* = 1), and cardiorespiratory failure due to pneumonia (*n* = 1). The composite outcome; PICU transfer and/or death occurred in a total of 23 (2.7%) admissions (equivalent to 23 children). For 806 (93%) ED encounters, the total hospital length of stay was known, of which 179 (22.2%) patients were admitted during 7 days or longer. Of 864 visits, data on temperature was obtained in 855 (99%) patients (median time after ED entry 24 min (IQR 5-62), on heart rate in 784 (91%) patients (median time after ED entry 24 min (IQR 5-68), on systolic blood pressure in 269 (31%) patients (median time after ED entry 86 min (IQR 14-290), on respiratory rate in 676 (78%) patients (median time after ED entry 29 min (IQR 6-100), on lactate in 39 (4.5%) patients (median time after ED entry 71 min (IQR 20-471), on white blood cell count in 663 (77%) patients (median time after ED entry 65 min (IQR 35-111), and on level of consciousness in 426 (49%) patients (median time after ED entry 34 min (IQR 9-135). qSOFA, SIRS, qPELOD-2, and qSOFA-lactate scores were positive for 40 out of 484 (8.3%), 415 out of 755 (55%), 11 out of 545 (2.0%), and 40 out of 151 (26.5%) visits, respectively (Figure 1).

### Performance qSOFA score

In patients with qSOFA ≥ 2, PICU transfer and/or mortality prevalence was 22.5% compared to 2.0% in patients with qSOFA < 2 (between group difference 20.5%, *p* < 0.01). Sensitivity, specificity, negative predictive value and positive predictive value of a positive qSOFA score for PICU transfer and/or mortality were respectively 50.0, 93.3, 98.0, and 22.5%. The positive qSOFA score AUROC for PICU transfer and/or death was 0.72 (95% CI, 0.57–0.86) and 0.53 (95% CI, 0.46–0.59) for prolonged hospital length of stay (Table 1).

**Table 1:** Prognostic accuracy of positive qSOFA, SIRS, qPELOD-2, and qSOFA-L scores for PICU transfer and/or mortality and prolonged hospital length of stay.

	**Primary outcome: PICU transfer and/or mortality**	**Secondary outcome: Prolonged hospital LOS (≥7 days)**	**Comparison to AUROC qSOFA positive**	
	**Area under the curve (95% CI)**	**Area under the curve (95% CI)**	***P*-value 1st outcome**	***P*-value 2nd outcome**
qSOFA positive	0.72 (0.57–0.86)	0.53 (0.46–0.59)	–	–
SIRS positive	0.64 (0.53–0.74)	0.49 (0.44–0.54)	0.23	0.82
qPELOD-2 positive	0.60 (0.45–0.76)	0.51 (0.45–0.57)	0.03	0.25
qSOFA-lactate positive	0.67 (0.50–0.84)	0.56 (0.46–0.67)	< 0.01	0.58
	**Primary outcome: PICU transfer and/or mortality**			
	**Sensitivity (%)**	**Specificity (%)**	**Negative predictive value (%)**	**Positive predictive value (%)**
qSOFA positive	50.0	93.3	98.0	22.5
SIRS positive	81.8	45.8	98.8	4.3
qPELOD-2 positive	22.2	98.7	97.4	36.4
qSOFA-lactate positive	58.3	76.3	95.5	17.5
	**Secondary outcome: Prolonged hospital length of stay (**≥**7 days)**			
	**Sensitivity (%)**	**Specificity (%)**	**Negative predictive value (%)**	**Positive predictive value (%)**
qSOFA positive	5.8	89.0	21.6	64.5
SIRS positive	55.0	47.7	22.2	79.6
qPELOD-2 positive	1.0	97.3	22.3	57.1
qSOFA-lactate positive	21.2	65.9	30.2	54.5
	**Prevalence PICU transfer and/or mortality**			
	**Positive score (%)**	**Negative score (%)**	**Between group difference (%)**	***P*****-value**
qSOFA	22.5	2.0	20.5	< 0.01
SIRS	4.3	1.2	3.1	0.010
qPELOD-2	36.4	2.6	33.8	< 0.01
qSOFA-lactate	17.5	4.5	13.0	0.009

The prognostic accuracy of the individual qSOFA components, systolic blood pressure, level of consciousness and respiratory rate, for PICU transfer and/or death were: AUROC 0.56 (0.39–0.74), 0.74 (0.58–0.90), and 0.54 (0.43–0.66), respectively. The prognostic accuracy of these individual qSOFA components for prolonged hospital length of stay were: AUROC 0.52 (0.44–0.60), 0.54 (0.47–0.61), and 0.50 (0.44–0.55), respectively (Table 2).

**Table 2:** Prognostic accuracy of the individual components of qSOFA for PICU transfer and/or mortality and prolonged hospital length of stay.

	**Primary outcome: PICU transfer and/or mortality**	**Secondary outcome: Prolonged hospital LOS (≥7 days)**
	**Area under the curve (95% CI)**	**Area under the curve (95% CI)**
qSOFA Systolic blood pressure positive^a^	0.56 (0.39–0.74)	0.52 (0.44–0.60)
qSOFA Level of consciousness positive^b^	0.74 (0.58–0.90)	0.54 (0.47–0.61)
qSOFA/SIRS Respiratory rate positive^c^	0.54 (0.43–0.66)	0.50 (0.44–0.55)
SIRS Temperature positive^d^	0.58 (0.45–0.70)	0.47 (0.42–0.51)
SIRS Leukocyte count positive^e^	0.49 (0.37–0.62)	0.54 (0.48–0.59)
SIRS Heart rate positive^f^	0.64 (0.51–0.76)	0.48 (0.43–0.53)

a Data on qSOFA variable systolic blood pressure was available for 269/864 visits: 10/269 were systolic blood pressure positive [2/10 PICU transfer and/or mortality, 4/8 prolonged hospital length of stay (2 unknown)], 259/269 were systolic blood pressure negative [11/259 PICU transfer and/or mortality, 63/230 prolonged hospital length of stay (29 unknown)].

b *Data on qSOFA variable level of consciousness was available for 426/864 visits: 47/426 were level of consciousness positive [8/47 PICU transfer and/or mortality, 15/38 prolonged hospital length of stay (9 unknown)], 379/426 were level of consciousness negative [6/379 PICU transfer and/or mortality, 77/355 prolonged hospital length of stay (24 unknown)]*.

c *Data on qSOFA and SIRS variable respiratory rate was available for 676/864 visits: 522/676 were respiratory rate positive [18/522 PICU transfer/mortality, 104/487 prolonged hospital length of stay (35 unknown)], 154/676 were respiratory rate negative [3/154 PICU transfer and/or mortality, 31/140 prolonged hospital length of stay (14 unknown)]*.

d *Data on SIRS variable temperature was available for 855/864 visits: 302/855 were temperature positive [11/302 PICU transfer and/or mortality, 53/282 prolonged hospital length of stay (20 unknown)], 553/855 were temperature negative [11/553 PICU transfer and/or mortality, 124/517 prolonged hospital length of stay (36 unknown)]*.

e *Data on SIRS variable leukocyte count was available for 663/864 visits: 341/633 were leukocyte count positive [11/341 PICU transfer and/or mortality, 78/310 prolonged hospital length of stay (31 unknown)], 322/633 were leukocyte count negative [11/322 PICU transfer and/or mortality, 61/303 prolonged hospital length of stay (19 unknown)]*.

f *Data on SIRS variable heart rate was available for 783/864 visits: 222/783 were heart rate positive [12/222 PICU transfer and/or mortality, 38/108 prolonged hospital length of stay (14 unknown)], 561/783 were heart rate negative [10/561 PICU transfer and/or mortality, 116/520 prolonged hospital length of stay (41 unknown)]*.

### Performance qSOFA score compared to SIRS and qPELOD-2

**Figure 2:**
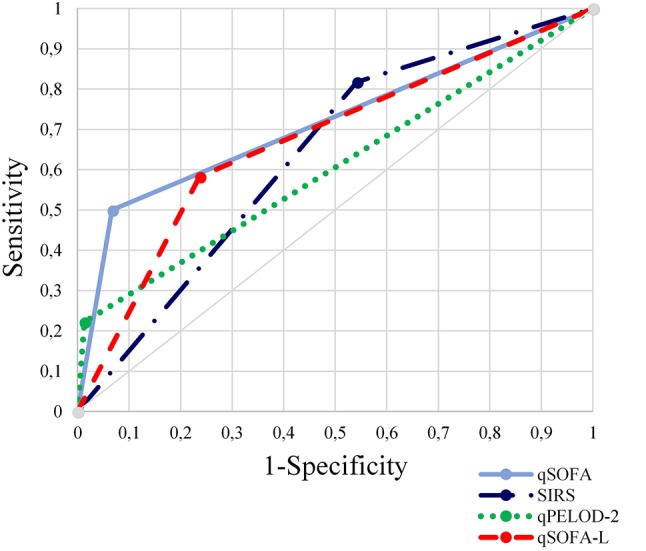
Comparison of area under the receiver operating characteristics curves for qSOFA, SIRS, qPELOD-2, and qSOFA-L scores to discriminate primary outcome (PICU transfer and/or mortality).

The AUROC of a positive qSOFA score for predicting PICU transfer and/or mortality tends to be higher than SIRS (AUROC, 0.64 [0.53–0.74], *p* = 0.23) and was significantly higher than qPELOD-2 (AUROC, 0.60 [0.45–0.76], *p* = 0.03) (Figure 2). The AUROC of a positive qSOFA score for predicting prolonged hospital length of stay (0.53 [0.46–0.59]) was not comparable to SIRS (AUROC, 0.49 [0.44–0.54], *p* = 0.82) and qPELOD-2 (AUROC, 0.51 [0.45–0.57], *p* = 0.25) (Table 1). The prognostic accuracy of each individual SIRS component is presented in Table 2.

### Performance qSOFA-lactate

Addition of venous lactate as an extra component to the qSOFA score resulted in an AUROC of 0.67 (95% CI, 0.50–0.84) for predicting PICU transfer/death and 0.56 (95% CI, 0.46–0.67) for prolonged hospital length of stay (Figure 2). qSOFA-lactate AUROC was significantly lower than qSOFA AUROC for PICU transfer and/or mortality (*p* < 0.01) (Table 1).

## Discussion

This single-center retrospective study of 864 ED visits and subsequent admissions for suspected bacterial infection, shows that qSOFA is a moderate predictor of PICU transfer and/or mortality in children. A previous study on qSOFA performance in the pediatric ICU showed comparable moderate prognostic accuracy of qSOFA for mortality (13). The discriminatory capacity of qSOFA for prolonged hospital length of stay was poor. This is not surprising since the qSOFA score was not validated for hospital length of stay.

Although not significantly, the prognostic accuracy of qSOFA for PICU transfer and/or mortality tends to be higher than commonly used SIRS criteria. Our relatively small sample size and small number of adverse events probably have hindered this analysis. Therefore, larger studies in the near future should compare qSOFA with other scores. qPELOD-2 has been suggested as a reasonable alternative for qSOFA, since PELOD-2 was found to discriminate decently for mortality in a PICU population of children with suspected infection (17). However, the prognostic accuracy for PICU transfer and/or mortality was significantly higher for qSOFA than qPELOD-2 in our cohort.

To further improve predictive accuracy, we explored whether the addition of lactate would be beneficial. A recent study in children reported a strong and independent association between increased lactate levels and mortality risk in the PICU (21). Furthermore, lactate has been shown to predict pediatric sepsis severity and was suggested to have utility in early risk stratification (19, 22, 23). In our study, inclusion of lactate in the qSOFA score decreased discriminatory capacity for PICU transfer and/or mortality. It has to be taken into consideration that this analysis is largely limited by the small numbers of obtained venous lactate levels. Moreover, arterial and capillary lactate measurements were unknown.

It could be debatable whether the included qSOFA variables (blood pressure, respiratory rate, and altered mental state) are sufficient in predicting outcome for children with infections. When looking at the prognostic accuracy of the individual qSOFA variables, systolic blood pressure and respiratory rate were poor predictors for PICU transfer and/or death, while level of consciousness showed to have moderate prognostic accuracy, similar to the qSOFA score. Arterial hypotension is known to be a very late sign of pediatric sepsis with poor sensitivity (24, 25), suggesting that blood pressure is not suitable in early detection of patients at risk for poor outcome. Secondly, respiratory rate could be influenced by many non-infectious factors, such as pain and inconvenience. Thus, the blood pressure and respiratory rate variables included in qSOFA may be aspecific for children and we question whether these variables will result in an adequate bedside prediction tool. Possibly, an algorithm applied to larger datasets of children with suspected infection could identify other suitable variables, either individually (e.g., level of consciousness, lactate) or as an addition to the qSOFA score. For example, heart rate has been suggested to be superior to respiratory rate in predicting critical care requirement (26). This trend was also seen in our cohort. Future studies are therefore urgently needed to identify parameters which could be useful in predicting adverse outcome in the ED.

The results of our study need to be interpreted with caution because this study is limited by the small sample size, high percentage of missing (i.e., not obtained) data, and relatively small number of adverse events. Future multicenter studies in larger populations are needed to draw firmer conclusions. In children with (suspected) bacterial infections presenting to the ED, mortality ranges from 0 to 2.2% and PICU transfer from 5.7 to 42% (22, 27–29). In our cohort, mortality (0.7%) and PICU transfer (2.1%) was lower. The reason for this is unclear; possibly, our cohort from a non-academic (secondary care) hospital involves children with relatively milder illness severity as compared to academic hospitals. Another reason could be that the threshold to admit patients and start antibiotics is lower in this center compared to others. However, children suspected for bacterial infections are managed according to our national guidelines. Furthermore, a large proportion of data was not obtained. Because our ED triage system does not oblige assessment of systolic blood pressure and level of consciousness, we hypothesize that these parameters have not been obtained in children not appearing ill. Our cohort also includes children with viral infections, resulting from our definition of suspected bacterial infection as initiation of antibiotic therapy within 24 h after ED entry without taking microbiology results into account. Ideally, prognostic scores should be evaluated in confirmed bacterial infections or in all febrile children visiting the ED. Another limitation of our study is that we were unable to adjust for comorbidities in the AUROC analysis, due to unknown data on patient history. Also, parameters (of score components) could have been measured at different times for different patients and since we monitored adverse outcomes during hospital stay, i.e., also past 24 h, the qSOFA score may not accurately reflect illness severity.

In conclusion, this is the first study to assess qSOFA criteria in a pediatric ED population. Since we compared qSOFA with other prognostic scores, our study contributes to current attempts to translate sepsis-3 criteria to children. qSOFA shows moderate prognostic accuracy for PICU transfer and/or mortality. The prognostic accuracy of qSOFA tends to be higher than SIRS and is significantly higher than qPELOD-2. Prognostic accuracy of qSOFA did not improve after inclusion of lactate. Prospective multicenter studies in larger ED populations of febrile children should be performed to further determine the utility of the qSOFA score in the pediatric ED. Pediatric sepsis researchers should assure that pediatric Sepsis-3 criteria are applicable to ED patients as well.

## Availability of data and material

The dataset used and analyzed supporting the conclusions of this manuscript are available from the corresponding author on reasonable request.

## Author contributions

SN and NB made fundamental contributions to the conception and design of this study. SN conducted the analysis and drafted the manuscript. NB, RB, GD, JH and HW read, revised and approved the final manuscript.

### Conflict of interest statement

The authors declare that the research was conducted in the absence of any commercial or financial relationships that could be construed as a potential conflict of interest.
