# Supplementary material for: Translating Sepsis-3 Criteria in Children: Prognostic Accuracy of Age-Adjusted Quick SOFA Score in Children Visiting the Emergency Department With Suspected Bacterial Infection
Source: Front Pediatr. 2018 Oct 1;6:266. doi: 10.3389/fped.2018.00266 (PMC6174358; doi:10.3389/fped.2018.00266)
Supplement: Supplementary file 1 [file Table_1.DOCX]

Supplementary Material

**Translating Sepsis-3 Criteria in Children: Prognostic Accuracy of Age-adjusted Quick SOFA Score in Children Visiting the Emergency Department with Suspected Bacterial Infection**

Sietske C. van Nassau^1^*, Ron H. van Beek^1^, Gertjan J. Driessen^2^, Jan A. Hazelzet^3^, Herbert M. van Wering^1^, Navin P. Boeddha^1,4,5^

1. Department of Pediatrics, Amphia Hospital, Breda, The Netherlands

2. Department of Pediatrics, Juliana Children's Hospital/Haga Teaching Hospital, The Hague, The Netherlands

3. Department of Public Health, Erasmus MC, University Medical Center Rotterdam, Rotterdam, The Netherlands

4. Intensive Care and Department of Pediatric Surgery, Erasmus MC-Sophia Children’s Hospital, University Medical Center Rotterdam, Rotterdam, The Netherlands

5. Department of Pediatrics, Division of Pediatric Infectious Diseases & Immunology, Erasmus MC-Sophia Children’s Hospital, University Medical Center Rotterdam, Rotterdam, The Netherlands

*** Correspondence:**Sietske C. van Nassau
Sietskevannassau@hotmail.com

# Supplementary Table

Supplementary table 1: Age-adjusted sepsis scores

| **Quick Sequential Organ Failure Assessment (qSOFA)**(1) | | Score | |
| --- | --- | --- | --- |
|  | **Age** | **0** | **1** |
| *1. Respiratory rate* | 0 days-1 week | ≤ 50 | >50 |
|  | 1 week-1 month | ≤ 40 | >40 |
|  | 1 month-1 year | ≤ 34 | >34 |
|  | 2 -5 years | ≤ 22 | >22 |
|  | 6-12 years | ≤ 18 | >18 |
|  | 13-17 years | ≤ 14 | >14 |
| *2. Altered mentation*  *AVPU or Pediatric GCS* | 0 days-18 years | A | V, P, U |
|  | 0 days-18 years | 15 | <15 |
| *3. Systolic Blood Pressure* | 0 days-1 week | ≥ 59 | <59 |
|  | 1 week-1 month | ≥ 79 | <79 |
|  | 1 month-1 year | ≥ 75 | <75 |
|  | 2 -5 years | ≥ 74 | <74 |
|  | 6-12 years | ≥ 83 | <83 |
|  | 13-17 years | ≥ 90 | <90 |
|  | | | |
| **Systemic Inflammatory Response Syndrome (SIRS)**(2, 3) | | Score | |
|  | **Age** | **0** | **1** |
| *1. Heart rate* | 0 days-1 week | 100-180 | >180 or <100 |
|  | 1 week-1 month | 100-180 | >180 or <100 |
|  | 1 month-1 year | 90-180 | >180 or <90 |
|  | 2-5 years | <140 | >140 |
|  | 6-12 years | <130 | >130 |
|  | 13-17 years | <110 | >110 |
| *2. Respiratory rate* | 0 days-1 week | <50 | >50 |
|  | 1 week-1 month | <40 | >40 |
|  | 1 month-1 year | <34 | >34 |
|  | 2-5 years | <22 | >22 |
|  | 6-12 years | <18 | >18 |
|  | 13-17 years | <14 | >14 |
| *3. Leukocyte Count (*10^3^/mm^3^)* | 0 days-1 week | <34 | >34 |
|  | 1 week-1 month | 5-19.5 | >19.5 or <5 |
|  | 1 month-1 year | 5-17.5 | >17.5 or <5 |
|  | 2-5 years | 6-15.5 | >15.5 or <6 |
|  | 6-12 years | 4.5-13.5 | >13.5 or <4.5 |
|  | 13-17 years | 4.5-11 | >11 or <4.5 |
| *4. Temperature* | 0 days-18 years | 36-38.5 | >38.5 or <36 |
|  | | | |
| **Quick Pediatric Logistic Organ Dysfunction 2 (qPELOD-2)**(4, 5) | | Score | |
|  | **Age** | **0** | **1** |
| *1. Hypotension*  *SBP or MAP* | 0 days-1 month | SBP>65 or MAP>46 | SBP<65 or MAP<46 |
|  | 1-11months | SBP>75 or MAP>55 | SBP<75 or MAP<55 |
|  | 12-23 months | SBP>85 or MAP>60 | SBP<85 or MAP<60 |
|  | 24-59 months | SBP>85 or MAP>62 | SBP<85 or MAP<62 |
|  | 60-143 months | SBP>85 or MAP>65 | SBP<85 or MAP<65 |
|  | ≥144 months | SBP>95 or MAP>67 | SBP<95 or MAP<67 |
| *2. Heart rate* | <12 years | <195 | >195 |
|  | ≥12 years | <150 | >150 |
| *3. Altered mentation*  *Pediatric GCS* | 0 days-18 years | >11 | <11 |
|  | | | |
| **Quick Sequential Organ Failure Assessment with Lactate (qSOFA-L)** | | Score |  |
|  | **Age** | **0** | **1** |
| *Criteria 1, 2 and 3 identical to above mentioned qSOFA score* | |  |  |
| *4. Lactate* | 0 days-18 years | <2mmol/L | ≥2 mmol/L |

**References**

1. Schlapbach LJ, Straney L, Bellomo R, MacLaren G, Pilcher D. Prognostic accuracy of age-adapted SOFA, SIRS, PELOD-2, and qSOFA for in-hospital mortality among children with suspected infection admitted to the intensive care unit. Intensive Care Med. 2018;44(2):179-88.

2. Goldstein B, Giroir B, Randolph A, International Consensus Conference on Pediatric S. International pediatric sepsis consensus conference: definitions for sepsis and organ dysfunction in pediatrics. Pediatr Crit Care Med. 2005;6(1):2-8.

3. Gebara BM. Values for systolic blood pressure. Pediatr Crit Care Med. 2005;6(4):500; author reply -1.

4. Leclerc F, Duhamel A, Deken V, Grandbastien B, Leteurtre S, Groupe Francophone de Reanimation et Urgences P. Can the Pediatric Logistic Organ Dysfunction-2 Score on Day 1 Be Used in Clinical Criteria for Sepsis in Children? Pediatr Crit Care Med. 2017;18(8):758-63.

5. Leteurtre S, Duhamel A, Salleron J, Grandbastien B, Lacroix J, Leclerc F, et al. PELOD-2: an update of the PEdiatric logistic organ dysfunction score. Crit Care Med. 2013;41(7):1761-73.

**
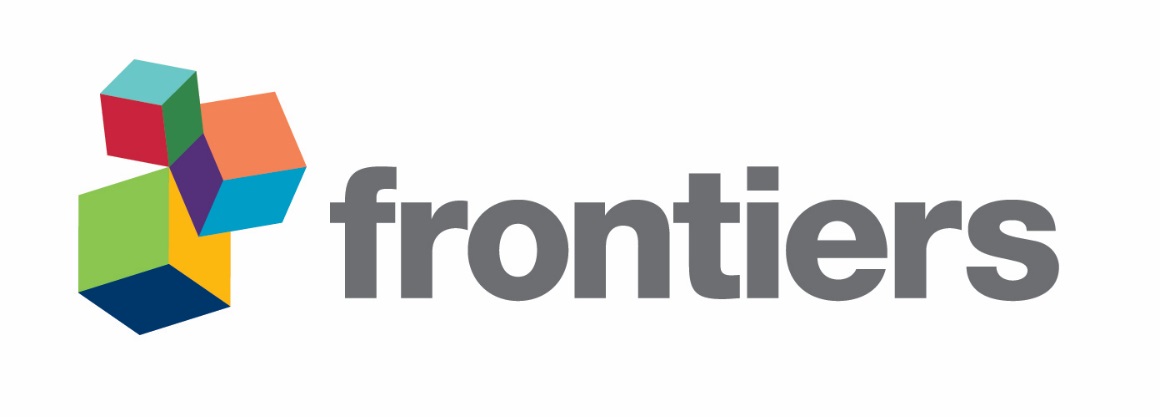
**
